# GENDERED RACIAL DISPARITIES IN FUNCTIONAL LIMITATIONS: EXPLORING THE ROLE OF LIFE-COURSE FAMILY CARE

**DOI:** 10.1093/geroni/igae098.0523

**Published:** 2024-12-31

**Authors:** Chioun Lee, Soojin Park

**Affiliations:** University of California Riverside, Riverside, California, United States; University of California Riverside, Riverside, California, United States

## Abstract

**Objectives:**

This study addresses the dearth of research on culturally embodied factors influencing health disparities, focusing on cultural aspects such as family formation timing, living arrangements, familism, and family care responsibilities. By employing “intersectionality” and a life-course approach to “family care,” we aim to understand why minority women are vulnerable to functional limitations.

**Methods:**

Data come from the MIDUS II, Refresher, and Milwaukee surveys, with a sample of Non-Hispanic Whites and Blacks (N=8,848; 18% Blacks; 53% Women). Measures of family care included age at first childbirth, number of biological children, single or nonmarital parenthood, child loss, co-residence with a disabled child, family caregiving history, and major grandparenting responsibilities. We identified racial disparities in family care profiles and assessed their contribution to health disparities.

**Results:**

Adverse family care profiles were more common among women, particularly Blacks. Compared to White women, Black women were more likely to experience early and non-marital motherhood, current family care, and major grandparenting roles. Adverse family care profiles were linked to more functional limitations, with differing effects between Black and White women. While early childbearing was more detrimental to White women, caring for disabled family members had a greater adverse effect on Black women. Adverse family care profiles explained one-third of Black-White disparities in functional limitations.

**Discussion:**

Our findings highlight the social costs of family care, disproportionately affecting minority women. They offer insights into public health policies and interventions aimed at easing the burdens of family care to improve their quality and length of life.

